# Supplementary material for: Novel Breast Cancer Brain Metastasis Patient-Derived Orthotopic Xenograft Model for Preclinical Studies
Source: Cancers (Basel). 2020 Feb 14;12(2):444. doi: 10.3390/cancers12020444 (PMC7072242; doi:10.3390/cancers12020444)
Supplement: Supplementary file 1 [file cancers-12-00444-s001.zip › cancers-683802-Supplementary materials.pdf]

Supplementary Materials: Novel Breast Cancer Brain Metastasis Patient-Derived Orthotopic Xenograft Model for Preclinical Studies

Masanori Oshi, Maiko Okano, Aparna Maiti, Omar M. Rashid, Katsuharu Saito, Koji Kono, Ryusei Matsuyama, Itaru Endo and Kazuaki Takabe

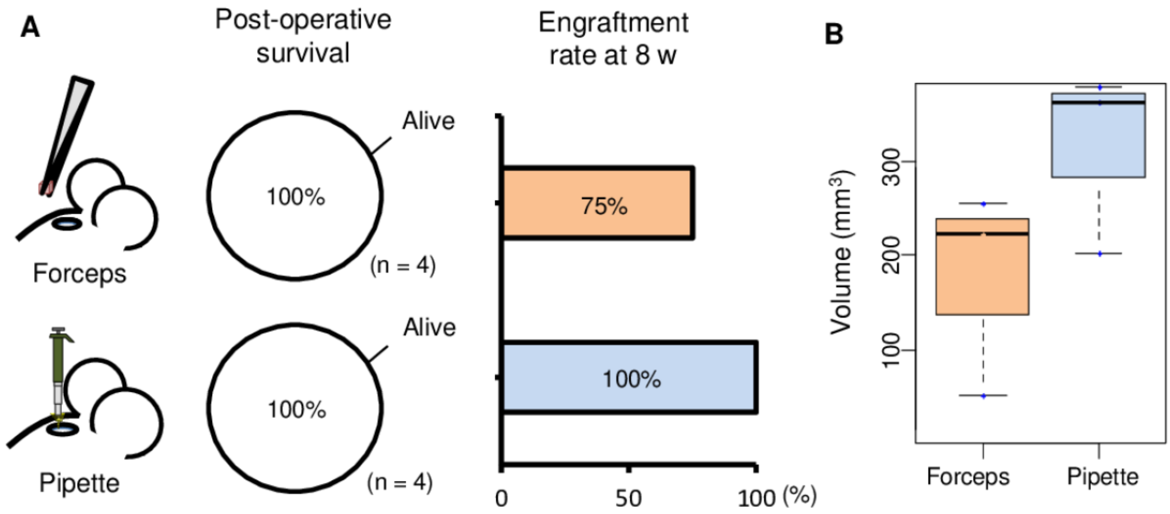

Figure 1. Comparison of two methods for tumor implantation in mouse brain. Brain metastasis of one estrogen receptor-positive breast cancer that had been passaged three passages as brain PDX was used for this experiment. The forceps and pipette methods were used for implantation of 1 μl tumor in mouse brain. (**A**) Pie charts depict survival rates within 1 day of implantation with the two methods. Rates of engraftment of implanted tumor after 8 weeks of surgery for tumor implantation are plotted for the two implantation methods. (**B**) Tukey boxplots of tumor volumes at 6 weeks in mice with engraftment are shown for the forceps and pipette methods. Magnetic resonance imaging was used for tumor volume measurement.

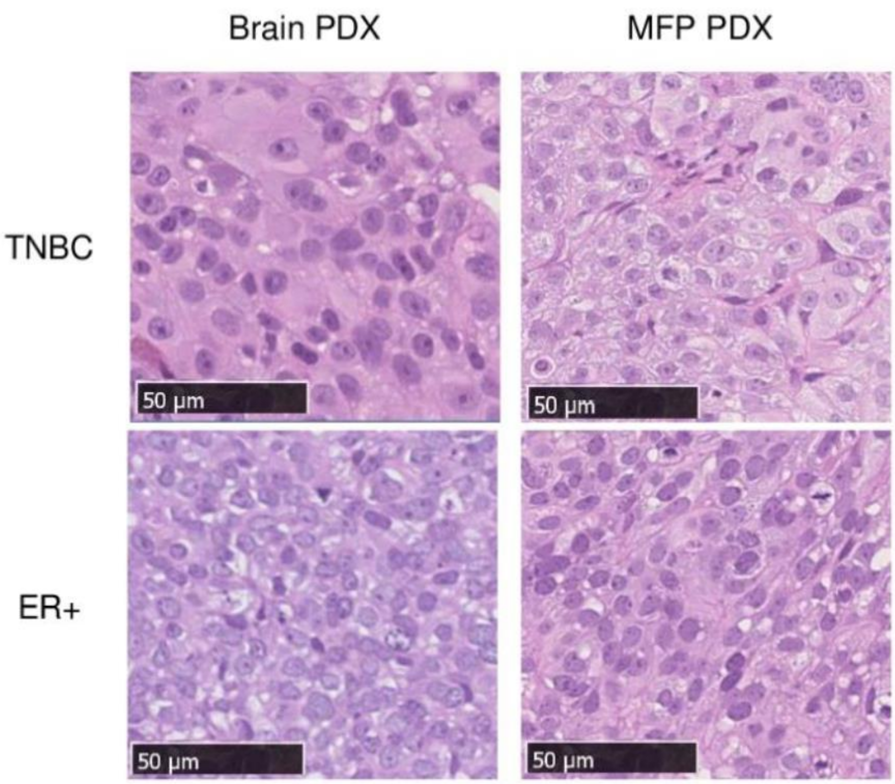

Figure S2. Histology of breast cancer brain metastases grown as xenografts in mouse brain or mammary fat pad (MFP). Hematoxylin-eosin-stained sections of patient-derived xenografts (PDXs) are shown for tumors from two patients with estrogen receptor-positive (ER+) or triple negative breast cancer (TNBC) or breast cancer. Scale bar = 50 μm.

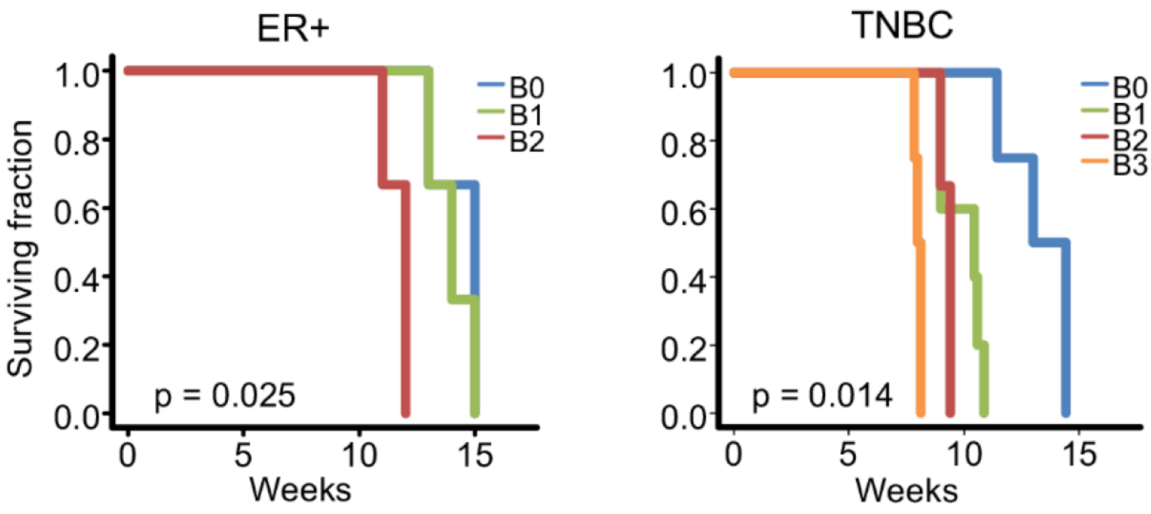

Figure S3. Survival of mice whose brains were implanted with brain metastasis of two breast cancer patients. Brain metastases of one each of estrogen receptor-positive (*ER+*) and triple negative breast cancer (*TNBC*) tumors were passaged thrice in mouse mammary fat pads and then implanted in brain (*B0*). Mice were sacrificed when symptom-based criteria were fulfilled, and their brain xenografts were further passaged 2–3 times through brain implantation in a similar manner (*B1–B3*). Shown are Kaplan-Meier survival plots with Cox regression analysis-based *p* values in comparison of survival of B0 and B2 mice.

Table 1. Human breast cancer brain metastasis samples used in this study.

	Patient		
	**#5**	**#10**	**#30**
Age at diagnosis (years)	37	48	29
Pathologic stage of primary tumor at diagnosis	2A	2B	1A
Other metastasis	Lung, lymph node	Skin	Lymph node
Overall survival (months)	49	35	38
Disease free survival (months)	42	28	28
Adjuvant therapy	Yes	Yes	Yes
Neoadjuvant chemotherapy	Yes	No	Yes
Estrogen receptor positivity of primary tumor	Negative	Positive	Negative
HER2 receptor positivity of primary tumor	Negative	Negative	Negative
Progesterone receptor positivity of primary tumor	Negative	Negative	Negative
Figures for which sample was used	S3	S1, S2, S3	1–6, S2

Table 2. Gene expression data generated in this study.

Microsoft Excel file Table_S2.xlsx.

Transcripts per million (TPM) values generated as described in the test are provided. Human Gene Nomenclature Committee gene symbols are used.

© 2020 by the authors. Licensee MDPI, Basel, Switzerland. This article is an open access article distributed under the terms and conditions of the Creative Commons Attribution (CC BY) license (http://creativecommons.org/licenses/by/4.0/).
